# Sustained PI3K Activation exacerbates BLM-induced Lung Fibrosis via activation of pro-inflammatory and pro-fibrotic pathways

**DOI:** 10.1038/srep23034

**Published:** 2016-03-14

**Authors:** Julia Barbara Kral, Mario Kuttke, Waltraud Cornelia Schrottmaier, Birgit Birnecker, Joanna Warszawska, Christina Wernig, Hannah Paar, Manuel Salzmann, Emine Sahin, Julia Stefanie Brunner, Christoph Österreicher, Sylvia Knapp, Alice Assinger, Gernot Schabbauer

**Affiliations:** 1 Institute of Physiology, Center for Physiology & Pharmacology, Medical Univ. Vienna, Austria; 2 CEMM, Research Center for Molecular Medicine of the Austrian Academy of Sciences, Vienna, Austria; 3 Department of Medicine I, Laboratory of Infection Biology, Medical Univ. Vienna, Austria

## Abstract

Idiopathic pulmonary fibrosis (IPF) is a life-threatening disease with limited treatment options. Additionally, the lack of a complete understanding of underlying immunological mechanisms underscores the importance of discovering novel options for therapeutic intervention. Since the PI3K/PTEN pathway in myeloid cells influences their effector functions, we wanted to elucidate how sustained PI3K activity induced by cell-type specific genetic deficiency of its antagonist PTEN modulates IPF, in a murine model of bleomycin-induced pulmonary fibrosis (BIPF). We found that myeloid PTEN deficient mice (PTEN^MyKO^), after induction of BIPF, exhibit increased TGF-β1 activation, mRNA expression of pro-collagens and lysyl oxidase as well as augmented collagen deposition compared to wild-type littermates, leading to enhanced morbidity and decreased survival. Analysis of alveolar lavage and lung cell composition revealed that PTEN^MyKO^ mice exhibit reduced numbers of macrophages and T-cells in response to bleomycin, indicating an impaired recruitment function. Interestingly, we found dysregulated macrophage polarization as well as elevated expression and release of the pro-fibrotic cytokines IL-6 and TNF-α in PTEN^MyKO^ mice during BIPF. This might point to an uncontrolled wound healing response in which the inflammatory as well as tissue repair mechanisms proceed in parallel, thereby preventing resolution and at the same time promoting extensive fibrosis.

Idiopathic pulmonary fibrosis (IPF) is characterized by high mortality rates, but only limited treatment options are available. The lack of understanding of molecular mechanisms, aetiology and progression underlying IPF make it a life-threatening disease. Hence, there is an urgent need to identify new molecules as triggering factors with therapeutic potential.

According to current knowledge, fibrosis often develops due to an uncontrolled wound healing response^1^. The sequential phases of wound healing - injury, inflammation and tissue repair - are dysregulated during fibrosis. Instead of structural re-organization, tissue is progressively destroyed which leads to a loss of organ function^2^. Inflammation during fibrosis is a double-edged sword as a strong early inflammatory response is thought to promote fibrosis, whereas late onset inflammation instead inhibits the pro-fibrotic outcome^1^.Since the time point of onset of reparative inflammation is crucial for its effect, it is important to understand the detailed mechanism of the disease progression in order to design new interventional treatment strategies.

The most prominent factor known to promote fibrotic diseases is transforming growth factor-β1 (TGF-β1). It is mainly released by macrophages and mediates the activation of myofibroblasts, promotes collagen synthesis and inhibits extracellular matrix degradation^3^,^4^. Thus, inhibiting TGF-β1 reduces extracellular matrix component deposition^5^,^6^. The expression of TGF-β1 is also induced by the pro-fibroticcytokineIL-13 via its receptor IL4Rα but also via IL13Rα2^2^. Overexpression of IL-13 leads to increased collagen deposition and fibrosis^7^, while fibrosis is decreased byIL-13 deficiency^8^ or by antibodies blocking IL-13^9^.In contrast, to TGF-β1, IL-6andtumour necrosis factor-α (TNF-α) are pro-inflammatory cytokines, which also display pro-fibrotic activity.IL-6 is found in the serum of patients suffering from systemic sclerosis^10^ and in the bronchoalveolar lavage fluid (BALF) of patients with IPF^11^. Targeting IL-6 by means of antibodies reduces collagen deposition and leukocyte infiltration in BLM induced dermal fibrosis^10^ and via trans signalling IL-6 promotes collagen I synthesis in dermal fibroblasts^12^.

Recently, our group showed that myeloid deficiency of the phosphatase and tensin homolog (PTEN) leads to reduced expression of IL-6 in macrophages in response to LPS^13^,^14^,^15^,^16^. In addition, we could provide evidence for an increased expression of the M2 markers Arginase I and Stabilin-1 and therefore the capability of PTEN to modulate the activation state of macrophages^15^. In acute inflammatory models of lung infection PTEN-deficiency in myeloid cells promotes survival via increased phagocytosis of bacteria, reduced TNF-α release and increased IL-10 production^17^,^18^. More recently, Yue and colleagues could demonstrate that myeloid PTEN knockout decreases ischemia reperfusion injury in mice^19^.

However, it remains unclear, how alterations in PTEN and the phosphatidylinositol 3-kinase (PI3K) axis in myeloid cells modulate chronic inflammatory diseases such as fibrosis. By means of the bleomycin (BLM)-induced pulmonary fibrosis (BIPF) model we aimed to identify how the PI3K/PTEN pathway in myeloid cells influences the severity of fibrosis, the leukocyte recruitment to the site of injury and the activation phenotype of macrophages in chronic fibrosis. Additionally, we were interested which cytokines and M1/M2 signature molecules may contribute to these effects. To achieve these aims we investigated mice with cell-type specific gene deletion of *pten* in the myeloid lineage. These myeloid PTEN-deficient mice and wild-type littermates were challenged with the antibiotic BLM to induce pulmonary fibrosis. BLM is in clinically approved to treat carcinomas and skin tumours^20^, however, it provokes pulmonary fibrosis as an adverse effect, which limits its application^21^. We found that myeloid PTEN-deficiency, accompanied by increased PI3K pathway activation, dramatically increased morbidity and reduced the survival of mice suffering from BIPF. The myeloid PTEN-deficient (further denoted as PTEN^MyKO^) mice exhibited increased weight loss and elevated collagen deposition in the lung compared to their wild-type (WT) littermates. These effects can be mainly attributed to a lack of leukocyte recruitment, in particular macrophages. Furthermore, we observed augmented production of pro-fibrotic and pro-inflammatory mediators in PTEN^MyKO^ mice.

## Results

### Myeloid PTEN deficiency increases BLM induced lung fibrosis

**Figure 1:**
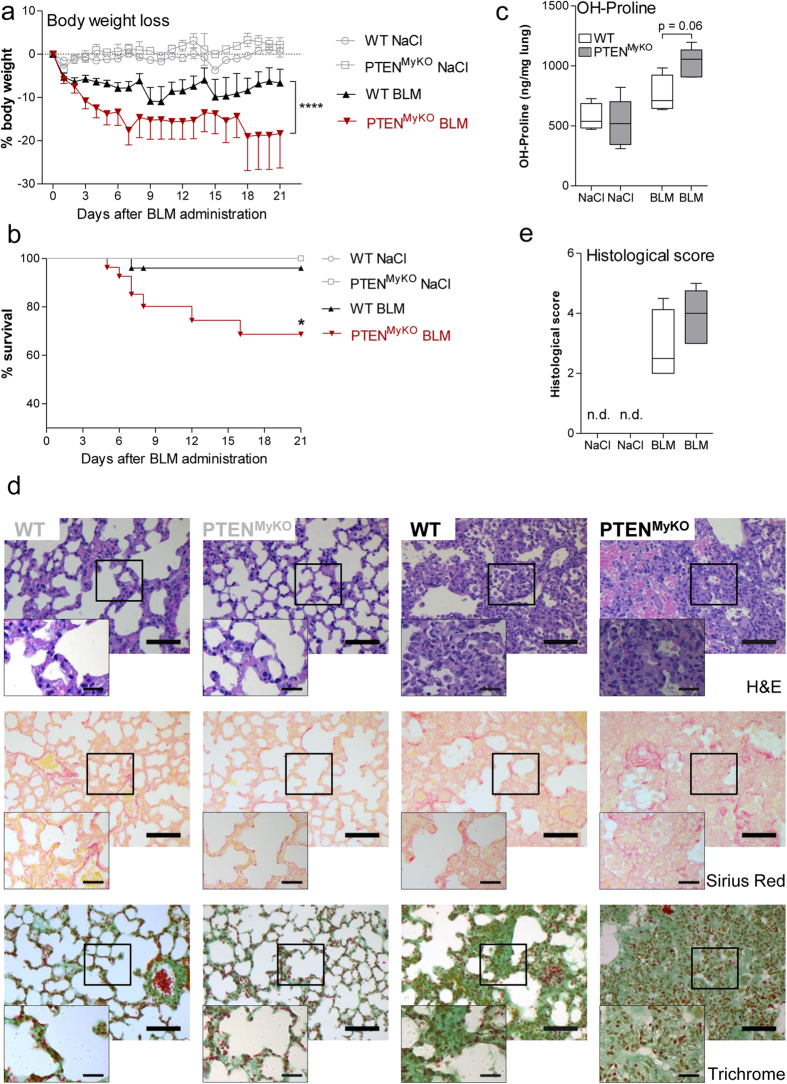
Myeloid PTEN deficiency exacerbates BLM induced pulmonary fibrosis. Myeloid PTEN deficient mice and wild-type littermates were treated with BLM (0.1 iu) intra-nasally. (**a**) The weight loss was assessed daily. Data are presented as mean +/− SEM and analysed by 2-way ANOVA, n = 19–20, ****p < 0.0001. (**b**) Myeloid PTEN deficiency led to a decreased survival. The mice were monitored over 21 days and the Kaplan Meier test was used for statistics, n = 20–24, *p < 0.05. (**c**) Hydroxyproline was quantified as measure for the collagen content in the lung after 21d and was statistically analysed by the Mann Whitney test, n = 4–5. (**d**) Representative images of healthy control lungs (labelled in gray) and BLM treated lungs (labelled in black) of a wild-type and a myeloid PTEN deficient mice (from left to right, 200× (large images) and 630× (small images) magnification). The lungs were stained with hematoxylin and eosin, Sirius red (collagen fibres stained red) and Masson’s Trichrome stain(muscle fibers = red, erythrocytes = orange, collagen  = green) (top-down), scale bars indicate 75 μm or 25 μm in the 200× or in the 630× magnification images, respectively. (**e**) The level of pulmonary fibrosis was scored according to the Sirius red staining of the histological lung specimen, n = 4–5.

The BIPF has facilitated the identification of factors and potential underlying mechanisms involved in the development of lung fibrosis. However, the complete pathogenesis still remains unclear and therapeutic interventions are missing. Since the deficiency of PTEN and consecutively enhanced PI3K activity in myeloid cells dampened the inflammatory response^14^ and in particular improved the survival of mice in an acute infectious model during pneumococcal pneumonia^17^, we wanted to investigate the role of the myeloid PI3K/PTEN axis in a model for chronic inflammation. Therefore, we challenged myeloid PTEN deficient mice and wild-type littermates with BLM (0.1 iu intra-nasally) for up to 21 days accompanied by a clinical assessment of the severity of disease. Myeloid PTEN knockout mice displayed a significantly enhanced loss of body weight (Fig. 1a). The weight changes were expressed as percentage of the weight of day zero, immediately before the administration of BLM. In addition to the increased morbidity, exemplified by the weight loss, we noted a significantly decreased survival (Fig. 1b) of PTEN^MyKO^ mice compared to their wild-type littermates. Increased mortality could also be observed when higher concentrations of BLM were administered (Supplemental Fig. I). The greatest decrease in weight was observed between d7 and d10 post BLM application. This coincides with the peak of initial inflammatory responses. Therefore we chose d7 as time point for further analyses to monitor inflammatory reactions in treated animals. While the wild-type mice almost recovered their initial body weight, the PTEN^MyKO^ mice did not gain weight again and even lost further weight after 18 days post BLM.

### Myeloid PTEN deficient mice exhibit increased collagen deposition

The main characteristic of fibrotic diseases is an excess of collagen deposition. The peak of collagen deposition is around 21 days after BLM administration^1^,^20^. Thus, this time point was chosen to analyse collagen deposition in the lungs of the treated mice. The amino acid hydroxyproline (OH-proline)is primarily found in collagen and is important for stabilizing its triple helix^22^. Therefore, OH-proline measurement is widely used to quantify collagen content in fibrotic tissues. We found that the lack of PTEN in myeloid cells increased collagen deposition in the lung in contrast to their wild-type littermates as measured by the increased levels of OH-proline (Fig. 1c). Additionally, we evaluated histological sections of pulmonary tissue. Haematoxylin and eosin staining indicated an increased pathologic reorganisation and a loss of the alveolar-capillary structure in the fibrotic lungs, which was even further exacerbated in myeloid PTEN-deficient mice (Fig. 1d, top panel). Collagen was visualized by Sirius red staining (Fig. 1d, middle panel) and Masson’s trichrome staining (Fig. 1d bottom panel). Analysis of lung sections using both stains indicated that the lack of myeloid PTEN enhanced pulmonary collagen deposition during BIPF (Fig. 1e).

### Expression of pro-fibrotic molecules is augmented in myeloid PTEN-deficient mice 7d post BLM instillation

**Figure 2:**
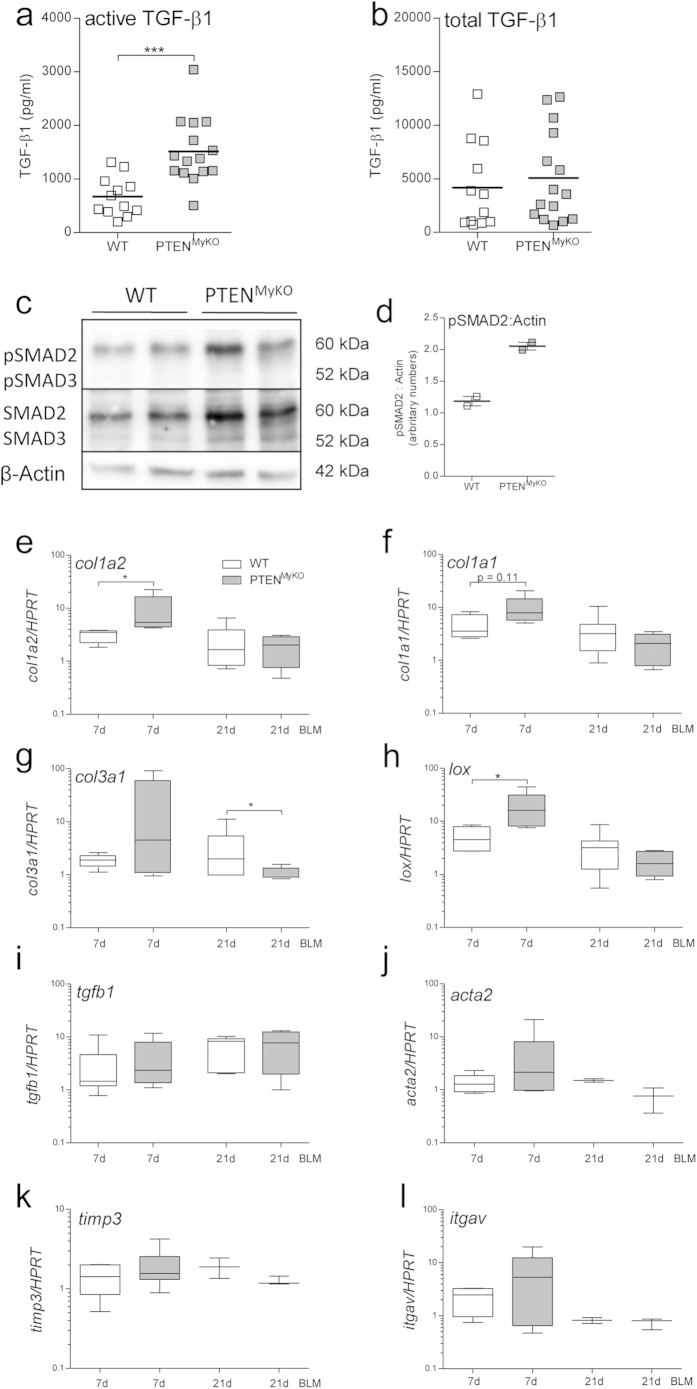
Active TGF-β1 and collagen expression are increased in PTEN^MyKO^ mice in early fibrosis. 7 days post BLM treatment the lungs of myeloid PTEN deficient and wild-type mice lungs were removed and homogenized either for ELISA and RNA isolation.(**a**)*In vivo* active (cleaved) TGF-β1 was measured by ELISA and found to be increased in myeloid PTEN deficient mice. (**b**) The same ELISA was performed after activating the samples with HCl *in vitro* for measuring latent TGF-β1 together with active TGF-β1. For statistical analysis the Student’s T-test was performed, n = 12–15, ***p < 0.001; (**c**)SMAD2/3 phosphorylation 7 days post BLM treatment. Broncho-alveolar lavage cells were analysed by Western blot. (**d**) Densitometric analysis of the protein content of the Western blot, the data are presented as fold of β-Actin and normalized to control mice, n = 2. (**e–l**) Lungs of BLM treated mice after 7d and 21d were homogenized for RNA isolation and qPCR was performed. The following genes were determined: *collagen, type I, alpha2* (*col1a2*), *collagen, type I, alpha 1* (*col1a1*), *collagen, type III, alpha1* (*col3a1*), *lysyl oxidase* (*lox*), *tgfb1*, *alpha smooth muscle actin* (a*cta2*), *tissue inhibitor of metalloproteinase 3* (t*imp3*), *integrin alpha V* (*itgav)*and *hypoxanthine guanine phosphoribosyl transferase* (*hprt*). Expression levels were normalized to HPRT as housekeeping gene. Data are shown as fold values of BLM treated wild-type (light bars) and PTEN^MyKO^ (grey bars) mice relative to treatment-naive mice. For statistical analysis the Mann Whitney test was performed, n ≥ 4 (7d), n ≥ 2 (21d), *p < 0.05

One of the most important growth factors and mitogens implicated in the development and progression of pulmonary fibrosis is TGF-ß1. TGF-ß1 has also been described to be involved in the transcriptional activation of a variety of pro-fibrotic molecules such as the collagen isoforms^23^. The common structural feature of collagens is a triple helix, formed by collagen alpha chains. The collagen type I is composed of the collagen alpha chain 1 (I) (col Iα1) and 2 (I) (col Iα2), and the collagen type III of the collagen alpha chain 1 (III) (col IIIα1)^24^.To analyse potential alterations of TGF-ß1 expression or activation we determined protein levels of the active form of TGF-ß1, which is derived after extracellular cleavage of the latent TGF-ß1. Additionally, we measured total TGF-ß1 in whole lungs of BLM treated animals. Consistent with our observation of enhanced collagen deposition and morbidity, we found significantly enhanced levels of the active form of TGF-ß1 in the PTEN^MyKO^ mice, whereas total protein was unaffected (Fig. 2a,b). This coincides with a comparable TGF-ß1 mRNA expression pattern (Fig. 2i). To identify possible downstream effects of active TGF-ß1, we analysed the phosphorylation of SMAD2 and SMAD3 in broncho-alveolar lavage cells of BLM treated mice. We found only a minimal increase in phosphorylated SMAD2 (Fig. 2c,d) in cells from myeloid PTEN deficient mice as compared to wild-type littermate mice. However, total SMAD2 was also slightly elevated. This increase might be mediated by miR-155, which is downregulated by AKT1^25^ and represses SMAD2 expression^26^.SMAD3 was not activated in either group during fibrosis (Fig. 2c).

We also isolated RNA from whole lung tissue on d7 and d21 post BIPF induction to determine the expression of pro-fibrotic markers such as the collagens *col Iα1* (*col Iα1*), *col Iα2* (*col Iα2*) and *col IIIα1* (*col IIIα1*). We found that *col Iα1* and *colIα2* mRNA expression levels were increased in mice 7d post BLM treatment compared to treatment-naive mice and that the expression was enhanced in the PTEN^MyKO^ mice. Interestingly, *collagen IIIα1* mRNA expression was only found augmented in the myeloid PTEN-lacking mice after BLM challenge (Fig. 2e–g). Cross-linking of the collagen fibrils is mediated by lysyl oxidase (LOX)^27^, which we found to display increased expression levels on d7 post BLM treatment as compared to control lung tissue. Again we observed significantly higher *lox* expression in the knockout mice as compared to littermate mice (Fig. 2h). However, at 21d post BLM treatment, the expression levels of the three procollagen α isoforms and of LOX had decreased again almost to the level of treatment-naive mice and no differences could be detected between the two genotypes. We did not observe variations in the expression of α-smooth muscle actin (acta2), which serves as a surrogate marker for fibroblasts or myofibroblasts (Fig. 2j). As already mentioned above, we could not detect differences in the mRNA expression of TGF-ß1 (Fig. 2i) and only modest, although not significant, upregulation of the αv-integrin, which also points at a possibility of increased conversion of latent TGF-ß to its active form (Fig. 2l). Additionally, we observed only slight and not significant changes in MMP3, 7, 8, 12 and 13 (data not shown) or TIMP3 expression (Fig. 2k), which indicates that degradation of extracellular matrix was not overly affected in the PTEN^MyKO^ mice.

### Characterization of pulmonary macrophages in PTEN^MyKO^ mice

Since PTEN^MyKO^ mice were severely affected by the BLM-induced lung fibrosis and the gene deficiency targets specifically monocytes and granulocytes but first and foremost macrophages, we intended to characterize macrophages in the lungs. To this end, we investigated a limited number of macrophage signature genes, which could shed some light on the polarization status of macrophages in dependence on the myeloid PTEN deficiency.

**Figure 3:**
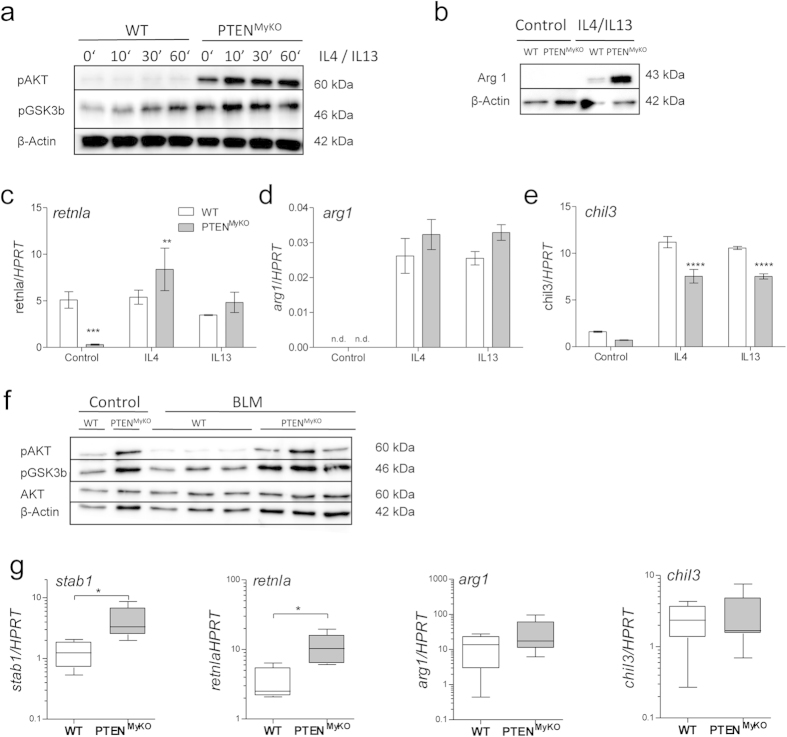
PTEN deficient macrophages display an enhanced M2- like phenotype *in vitro* and during fibrosis. Alveolar macrophages (AMs) were isolated from the lungs of myeloid PTEN knockout mice and wild-type littermates and stimulated with IL-4 (10 ng/ml), IL-13 (10 ng/ml) or both in combination. (**a**) AMs were stimulated with IL-4 and IL-13 for the indicated time points and phosphorylation of AKT and GSK3β was detected by Western Blot, which were increased in PTEN deficient macrophages. (**b**) IL-4/IL-13 stimulation overnight led to increased Arginase I (Arg1) expression in the macrophages lacking PTEN. (**c–e**) AMs were stimulated with IL-4 or IL-13 o/n, RNA was isolated and analysed by qPCR. The M2 markers Resistin like alpha (Retnla, FIZZ1), Arg1 and Chitinase-like 3 (Chil3, YM1) were determined. The Data were analysed by 2-way ANOVA, n = 4, **p < 0.01, ***p < 0.001, ****p < 0.0001. (**f**) Western Blot analysis of phospho-AKT, phospho-GSK3β, AKT and β-Actin of macrophages isolated from single cell suspension of lungs of wild-types and myeloid PTEN deficient mice 14d post BLM treatment and control mice. (**g**) Total lung of BLM treated mice was homogenized at d7 and RNA was isolated. Via qPCR the expression of the genes *st*a*bilin1* (*stab1*), *retnla*, *arg1* and *chil3* was measured. For statistical analysis the Mann Whitney test was performed, n ≥ 4, *p < 0.05. Expression levels were normalized to HPRT as housekeeping gene. Data are shown as fold values of BLM treated wild-type (light bars) and PTEN^MyKO^ (grey bars) mice relative to treatment-naive mice.

To verify the increased activation of the PI3K signalling axis upon PTEN deletion, we isolated alveolar macrophages and stimulated them with IL-4 and IL-13. Even at baseline we detected enhanced phosphorylation of AKT and downstream GSK3ßin PTEN deficient macrophages. This could not be further increased by the addition of IL-4 and IL-13 (Fig. 3a). When we analysed the M2 marker Arginase I, we found an enhanced expression *in vitro* in PTEN-deficient macrophages upon IL-4 and IL-13 stimulation by Western blot analysis (Fig. 3b), which could also be seen on a transcriptional level (Fig. 3d), although it did not reach statistical significance. Analyses of other important macrophage polarization signature genes revealed that in the PTEN^MyKO^ alveolar macrophages *retnla* (coding for Rtnlaalso known as Relmαor FIZZ1) was significantly upregulated upon stimulation (Fig. 3c). In contrast, *chil3* (coding for Chi3l3also known as YM-1) was significantly downregulated (Fig. 3e). Recently, similar results were observed in PTEN deficient bone marrow derived macrophages (BMDMs), which are the more common model for analysing macrophage polarization. We could show that PTEN deficiency promotes a shift towards alternative activation of BMDMs, indicated by increased Arginase I and Stabilin-1 expression^15^. To confirm this finding *in vivo* in BIPF we enriched the amount of macrophages from lungs by generating an adherent single cell suspension of the lung under cell culture conditions. We observed that PTEN-deficient macrophages from control mice had increased amounts of phosphorylated AKT and GSK-3ß (Fig. 3f), which was not further enhanced during fibrosis, indicated by a similar phosphorylation pattern of these proteins in macrophages obtained from BLM-treated mice at d14.

To investigate if the increased PI3K activity influences the macrophage polarization during pulmonary fibrosis, we investigated the expression of macrophage polarization genes in whole lung tissue on d7 post BLM treatment, as in the lung the expression of Stab1^28^, Retnla^29^, and Chil3^29^,^30^ are predominantly restricted to macrophages. In contrast, Arginase I is also constitutively expressed in bronchial epithelial cells, endothelial cells and (myo-) fibroblasts^31^.In line with our *in vitro* observations, when we analysed the M2 marker gene expression, we detected upregulation of Stabilin-1, Retnla and a trend for Arginase I in BLM-treated PTEN^MyKO^ mice compared to littermate wild-type controls, while Chi3l3 was not changed (Fig. 3g). However, due to the wide expression profile of Arginase I in lung cells, we cannot exclude that other cells apart from macrophages contribute to our observed levels. This indicates that beside the enhanced expression of pro-fibrotic TGF-ß1 as well as collagen expression, we could observe a shift to alternative activation of lung macrophages in the PTEN^MyKO^ mice.

### Reduced broncho-alveolar infiltration by myeloid and lymphoid cells in fibrotic PTEN^MyKO^ mice

**Figure 4:**
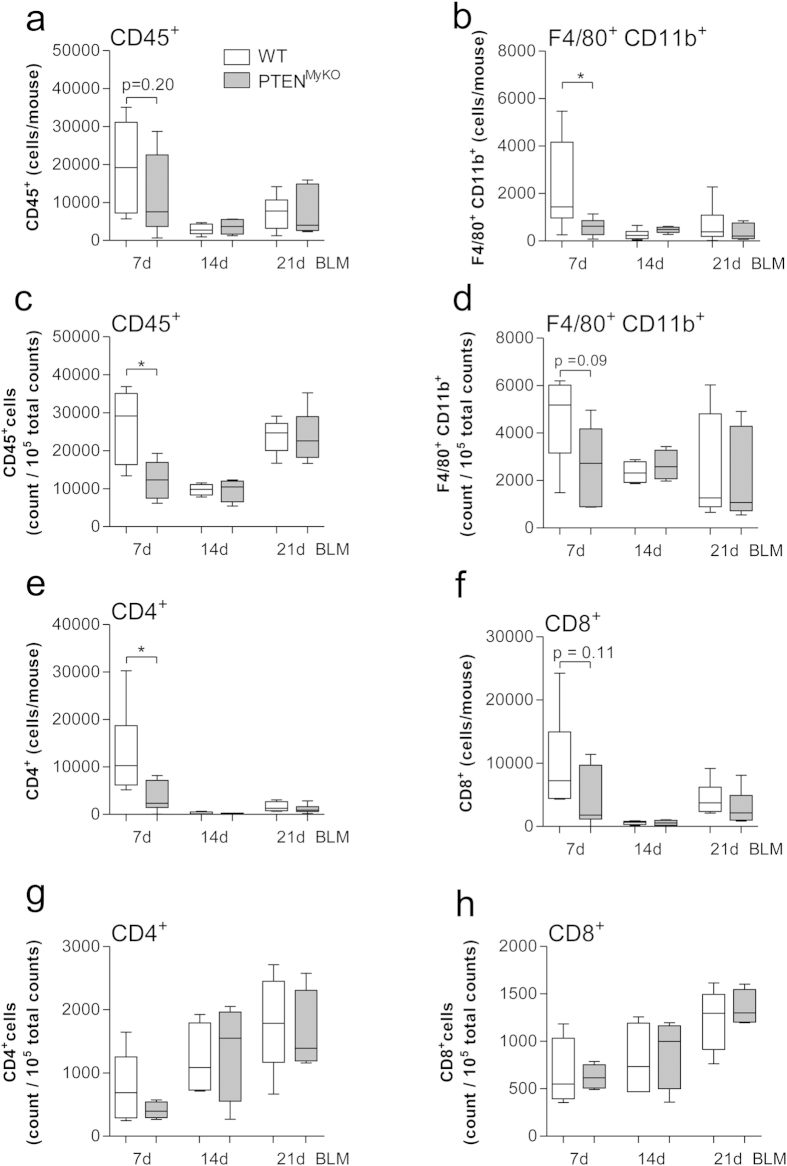
During pulmonary fibrosis extravasation of PTEN deficient macrophages is diminished. Flow cytometric analysis of BAL cells (**a,b,e,f**) or a single cell suspension of the total lung (**c,d,g,h**) was performed of wild-type mice and mice lacking myeloid PTEN 7d, 14d and 21d post BLM treatment. (**a,c**) leukocytes were identified by an α-CD45 antibody. (**b,d**) α-CD45, α-F4/80, α-CD11b antibodies were used to identify macrophages. T-cells were distinguished by an α-CD4 antibody (**e,g**) or α-CD8 (**f,h**) antibody to determine T helper cells and cytotoxic T cells, respectively. For statistical analysis the unpaired Student’s t-test (**a–g**) or the Mann Whitney test (**h**) was performed, n≥6, *p < 0.05.

Myeloid cells contribute to the severity of the pulmonary fibrosis, but are at the same time involved in the resolution of inflammation and tissue repair^32^. The deficiency of PTEN in myeloid cells exacerbated the disease progression in BIPF. Moreover, we observed changes in the polarization state of alveolar macrophages. Thus, we aimed to analyse the influx of these important inflammatory cells into the lungs of BLM-treated mice. We harvested the BALF 7d post BLM treatment and analysed the cell subset composition in the BALF (Fig. 4a,b,e,f) as well as whole lung tissue (Fig. 4c,d,g,h) of PTEN^MyKO^ mice by flow cytometry. At 7d post BLM treatment we found reduced numbers of CD45+ hematopoietic cells in both BALF and whole lung tissue, although the data failed to reach significance in the BALF (p = 0.20). At 14d post BLM treatment this influx declined in the BALF and whole lung, but rose again at d21. At this time point CD45+ counts did not reach levels of d7 again in either BALF or whole lung. No significant differences between wild-type and PTEN^MyKO^ mice could be observed at d14 or d21 (Fig. 4a,c).

Further cellular characterization revealed that at d7, CD45+ F4/80+ CD11b+macrophages are significantly reduced in the BALF of PTEN deficient mice (Fig. 4b). In whole lung tissue F4/80+ CD11b+ macrophages also showed a tendency to be diminished in BLM-treated PTEN^MyKO^ mice, but again data did not reach significance (p = 0.09) (Fig. 4d). In the BALF the amount of F4/80+ CD11b+ macrophages peaked at d7 and dropped afterwards to very low levels at d14 and d21. In contrast, analysis of the total lung revealed only a slight decline of F4/80+ CD11b+ macrophages after d7. No differences between genotypes could be observed after d7 in BALF or whole lung. Extravasation of other cell types such as Ly6G+ neutrophil granulocytes was not affected by the *pten* gene deficiency (data not shown). This indicates that numbers of infiltrating monocytes and macrophages are reduced in the early phase 7 days post BLM administration, which relates to the exacerbated clinical outcome of the disease. Analysis of lung lymphoid cells revealed significantly reduced numbers of CD4+ T-helper cells and a tendency toward fewer CD8+ cytotoxic T-cells in the BALF at d7 after BLM treatment in PTEN^MyKO^ mice (Fig. 4e,f). A similar trend for CD4+ and CD8+ T-cells could be observed in whole lungs (Fig. 4f,h). While in the BALF the number of CD4+ as well as CD8+ T-cells seemed reduced in PTEN^MyKO^ mice at d7, the cell counts in the total lung were unaltered by myeloid PTEN-deficiency. Numbers of CD4+ and CD8+ T-cells tended to rise continuously until d21, thereby likely contributing to the increased CD45+ cell counts observed at d21. In contrast, B-cell counts (data not shown) were unchanged in lungs 21d post BLM administration. Taken together, these data point towards a dysfunctional recruitment of myeloid as well as lymphoid cells in PTEN^MyKO^ mice upon treatment with BLM.

### Reduced re-stimulatory capacity of T-cells derived from BIPF PTEN^MyKO^ mice

To further investigate potential secondary effects of the myeloid PTEN deficiency on the activation and polarization of T-cells and the diminished presence of CD4+ and CD8+ T-cells in PTEN^MyKO^ mice, we performed experiments to monitor re-stimulation of T-cells in the lungs of mice subjected to BIPF. In other pathologic settings such as autoimmunity our group already observed a dysfunctional Th17 response in myeloid cell specific PTEN deficient mice in a murine model of multiple sclerosis. In this context T-cells were unable to produce Th17 signature cytokines such as IL-17A and IL-22^16^.

**Figure 5:**
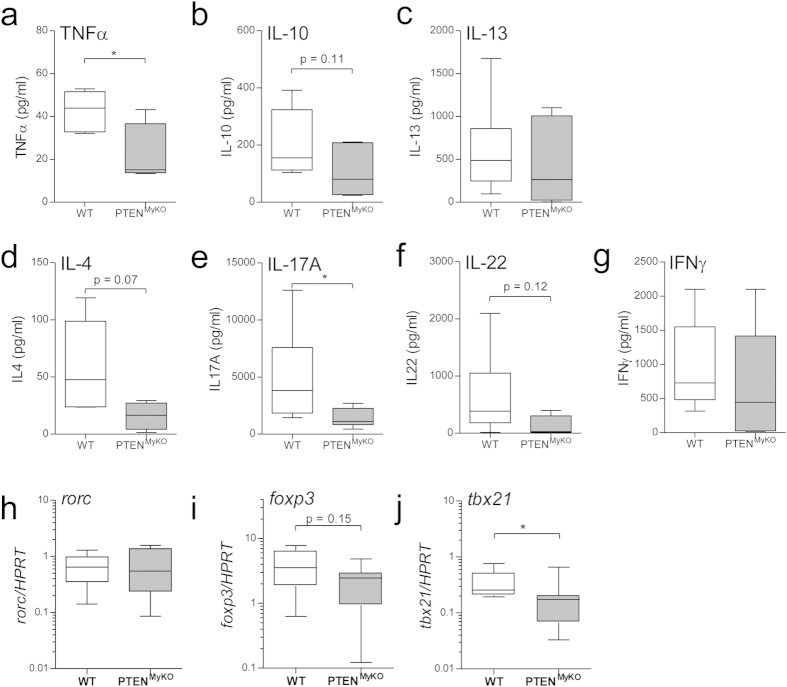
Decreased inflammatory response of T-cells derived from BLM treated myeloid PTEN KO mice compared to WT littermates. T-cells, isolated from mice 7d after instillation of BLM, were *ex vivo* re-stimulated with an α-CD3e antibody for 4 days and the cytokines TNF-α (**a**), IL-10 (**b**), IL-13 (**c**), IL-4 (**d**), IL-17A (**e**), IL-22 (**f**), INF-γ (**g**) were measured in the supernatant by ELISA. (**h–j**) RNA was isolated from lungs at day 7 of mice treated with BLM and analysed by qPCR for the mRNA expression of *il10*, *rorc*, *foxp3* and *tbx21*. Expression levels were normalized to HPRT as housekeeping gene. Data are shown as fold values of BLM treated wild-type (light bars) and PTEN^MyKO^ (grey bars) mice relative to treatment-naive mice. For statistical analysis the unpaired Student’s t-test (**a–c,e–I,k**) or the Mann Whitney test (**d,j**) was performed, n ≥ 6, *p < 0.05.

We therefore isolated whole lungs from BIPF mice (PTEN^MyKO^ and wild-type littermates) and prepared single cell suspensions, which were then re-stimulated *in vitro* with anti-CD3ε antibody for 4 days to trigger cytokine release. Next, the secreted cytokines in the cell-culture supernatants were measured by ELISA. In general, we found reduced T-cell cytokine production in cells, which were derived from fibrotic lungs of PTEN^MyKO^ mice. Most notably were the significant reductions of TNF-α and IL-17A secretion (Fig. 5a,e) and a trend for reduced IL-10, IL-4 and IL-22 (Fig. 5b,d,f). However, IL-13 and IFN-γ levels were found to be unaltered (Fig. 5c,g). Our data indicate that in addition to reduced T-cell accumulation in the injured lungs of PTEN^MyKO^ mice, T-cells, including Th1, Th2, Th17 and to a lesser extend Treg subsets, were far less responsive. Moreover, we analysed the transcription factor profile of T-cell specific transcription factors required for the polarization and activation. Interestingly, we found a trend for reduced expression of the Treg-TF Foxp3^33^ in the lungs of BLM treated PTEN^MyKO^ mice (Fig. 5i). Further, we could observe significant down-regulation of Tbet, also known as tbx21, identified to trigger Th1 cells^34^ in PTEN^MyKO^ mice (Fig. 5j), whereas RORγt, also known as rorc, also reported to induce Th17 cells^35^, was neither induced compared to treatment-naive mice nor differentially expressed between the genotypes (Fig. 5h). Altogether, we found an impaired T-cell response in PTEN^MyKO^ mice during BIPF.

### Lung cytokine expression in BIPF

**Figure 6:**
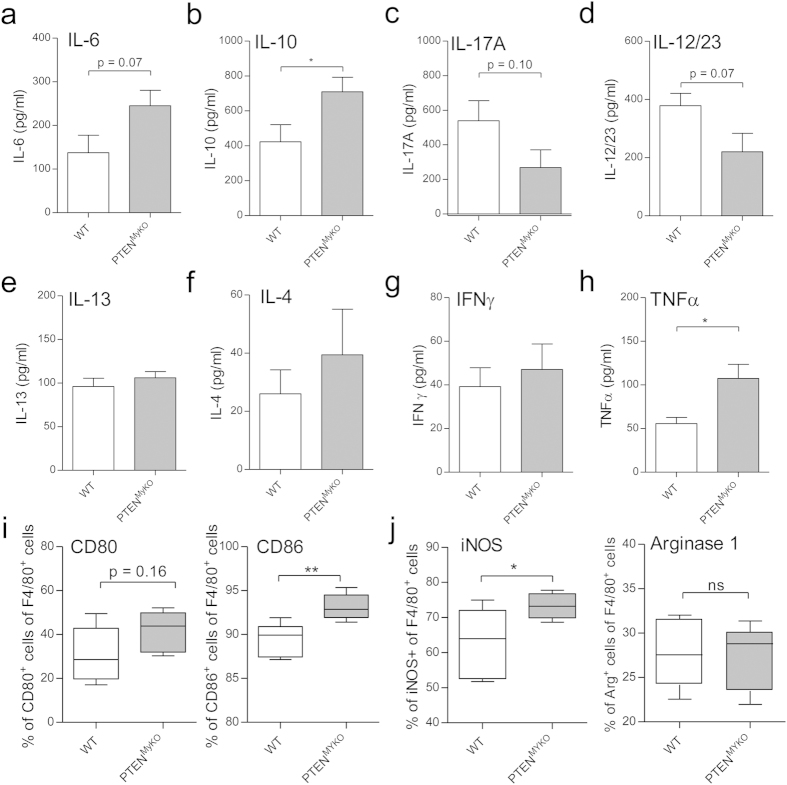
Pro-inflammatory cytokines are increased and macrophages display a shift towards an M1 phenotype. The cytokines IL-6 (**a**), IL-10 (**b**), IL-17A (**c**), IL-22/23 (**d**), IL-13 (**e**), IL-4 (**f**), INF-γ (**g**) and TNF-α (**h**) were measured by ELISA in homogenized lung tissue from wild-type and myeloid PTEN deficient mice at 7d post BLM treatment. For statistical analysis the unpaired Student’s t-test (**b,d**) or the Mann Whitney test (**a,c,e,h**) was performed, n ≥ 9, *p < 0.05. (**i**,**j**) Lungs from mice subjected to pulmonary fibrosis were digested by DNAse I and Collagenase I to generate a single cell suspension. Macrophages were identified by CD45^+^ and F4/80^+^ and further analysed for their expression of CD80, CD86 (**i**) and iNOS, ArgI (**j**). For statistical analysis the unpaired Student’s t-test was performed, n ≥ 5, *p < 0.05, **p < 0.01.

The expression and the release of a great variety of pro-fibrotic cytokines are known hallmarks of fibrosis. Pharmacological targeting these cytokines is currently under clinical investigation with drugs, such as pirfenidone, that acts on the activity of TGF-ß and is already clinically approved in the EU, Japan and USA. Further candidate drugs include ethanercept, inhibiting the TNF pathway and QAX576, a monoclonal antibody blocking IL-13 3. Therefore we aimed to investigate the expression profiles of several of the cytokines and mitogens implicated in the progression of pulmonary fibrosis at d7 post BLM treatment. Interestingly, we found an increased expression of IL-6 (Fig. 6a), significantly augmented release of IL-10 (Fig. 6b) and in particular active TGF-ß1 (Fig. 2a) in the PTEN^MyKO^ mice as compared to wild-type littermate control mice. IL-6 is a pleiotropic cytokine, which exhibits pro-fibrotic properties, such as promoting collagen I synthesis via TGF- ß1^12^. Besides its cytokine functions, IL-6 can also act as growth factor for myofibroblasts^36^.

The role of IL-10 is rather unclear in the progression of fibrosis. IL-10 is a potent anti-inflammatory molecule and thereby suppresses the action of many pro-inflammatory and pro-fibrotic molecules. In contrast, IL-10 can exert pro-fibrotic properties by supporting lymphocyte recruitment and Th2 respones^37^. The sources of IL-10 expression and secretion can be manifold. Myeloid cells can produce IL-10 but also regulatory T-cells are potent IL-10 producers and thereby prevent fibrosis^38^.

Furthermore, we measured T-cell cytokines and T-cell polarizing cytokines such as IL-17A, IL12/23 p40, IL-4, IL-13 and IFN-γ at seven days post BLM administration. We identified a trend for reduced amounts of IL-17A and IL-12/23 (Fig. 6c,d), which coincides with reduced numbers of T-cells in the injured lungs (Fig. 4e,f) and the reduced re-stimulatory capacity of T-cells in BIPF (Fig. 5a–g). IL-13, IL-4 and IFN-γ were not significantly different in the myeloid PTEN deficient mice subjected to pulmonary fibrosis (Fig. 6e–g). TNF-α, which is produced by myeloid but also lymphoid cells and is considered to be a potent pro-inflammatory and pro-fibrotic mediator, was significantly upregulated in the PTEN^MyKO^ mice as compared to wild-type littermate control mice (Fig. 6h). Our data support the idea that differential release or activation of cytokines and mitogens drive the pro-fibrotic phenotype observed in the PTEN^MyKO^ mice. Since we discovered augmented levels of IL-6 and TNF-α, which are cytokines characteristic for M1 macrophages, and no changes in the release of IL-4 and IL-13, the signature molecules of M2 macrophages, we analysed surface and intracellular markers for macrophage polarization via flow cytometry. Surprisingly, we detected that a greater percentage of CD45+ F4/80+ macrophages of the lungs of PTEN^MyKO^ mice express the M1 markers CD86 (p = 0.006) and CD80 (p = 0.161) (Fig. 6i) on their surface and iNOS intracellularly (p = 0.040) (Fig. 6j), but there was no difference in the expression of intracellular Arginase I compared to wild-type littermates. While we found a slight increase in the mRNA expression of Arginase I in total lung tissue in PTEN^MyKO^ mice compared to wild-type littermates (Fig. 3g), we did not detect an increase in CD45+ F4/80+ macrophages positive for this M2 marker (Fig. 6j). This increase in Arginase I could therefore derive from other cell types, including (myo)-fibroblasts or endothelial and epithelial cells. This result somewhat contradicts our previous finding that the deficiency of PTEN shifts macrophages towards an alternative phenotype (Fig. 4). A possible explanation is that during fibrosis the inflammatory phase and the tissue repair phase of the wound healing process coincide and as both phenotypes of the macrophages are more pronounced in the PTEN^MyKO^ mice, also the detrimental effects are exaggerated leading to augmented collagen expression and deposition.

## Discussion

In this study we show that PTEN deficiency in myeloid cells drives the development and the progression of pulmonary fibrosis induced by the administration of BLM. We noted increased weight loss and reduced survival during BIPF, which was due to a massive reorganization and collagen deposition in the lungs of PTEN^MyKO^ mice. Most of the altered parameters that we observed during BLM treatment, such as the lack of leukocyte recruitment, were apparent after d7 in the myeloid PTEN-deficient mice. These changes point at a dysregulated early inflammatory response, which then translates into an exacerbated chronic phase resulting in increased morbidity and mortality.

In this study we focused on effects of *pten*-deficiency in myeloid cells by applying a genetic approach specifically targeting the myeloid lineage, in particular monocytes, macrophages but also granulocytes and some dendritic cell subsets become genetically modified. This lack of PTEN resulted in reduced extravasation of F4/80+ CD11b+ macrophages into the lung during pulmonary fibrosis compared to wild-type mice. This supports previous reports showing that PTEN is crucial for the recruitment of inflammatory cells (such as neutrophil granulocytes) to the site of inflammation^39^. However, a recent publication using conventional PI3Kγ-knockout mice in BLM-induced lung fibrosis could show reduced monocyte and macrophage cell numbers in the BAL on d16 post treatment^40^. Nevertheless, we only found significantly changed numbers of recruited macrophages 7 days post BLM. Investigations of the cell influx at d14 and d21 revealed that numbers of macrophages started to decline compared to d7, while no differences were observed between the two genotypes. Taken together, these findings indicate that tight regulation of the PI3K signalling pathway is required for normal migratory behaviour of inflammatory cells in BIPF.

Histological assessment of lung specimen indicated increased inflammation and cell influx in PTEN^MyKO^ mice in comparison to wild-types due to H&E staining 21d post BLM treatment. However, when we addressed this question more precisely by flow cytometry we did not find any significant changes in leukocytes numbers between PTEN^MyKO^ mice and wild-types 21d post BLM treatment. Since we did not determine fibroblast and fibrocyte recruitment by flow cytometry, we can only speculate whether these cell types are more abundant at later stages of BIPF.

However, we noticed increased production of the pro-fibrotic cytokines IL-6, TNF-α and IL-10, although the role for IL-10 in fibrosis is still controversial. IL-6 is an important mediator of fibrosis and is stimulated by TNF-α. IL-6 acts as a mitogen on fibroblasts and myofibroblasts and thereby promotes scar formation^36^,^41^. Further, it promotes collagen I synthesis and TGF-β signalling^12^. TNF-α acts pro-fibrotic and is directly involved in BLM induced lung fibrosis^42^. A number of TNF-targeted therapies to treat pulmonary fibrosis are currently under clinical investigation. F4/80+ CD11b+ inflammatory macrophages can produce large amounts of pro-fibrotic cytokines (such as TNF-α and IL-6), which is even more pronounced in the PTEN^MyKO^ mice. Nevertheless, we cannot exclude that other cell types (such as fibrocytes or fibroblasts) contribute to this pro-inflammatory cytokine milieu. It has been recently shown that depletion of macrophages in an early phase of fibrosis is protective in a model for liver fibrosis. In contrast, low numbers of macrophages during the resolution phase lead to reduced matrix degradation and more fibrosis^43^.Thereby, macrophages seem to be directly involved in the pathogenesis of pulmonary fibrosis. In patients with IPF, increased numbers of M2 macrophages are found^44^, which is associated with increased fibrosis^45^. The fibrotic process is also influenced by M1 macrophages, which secrete pro-inflammatory cytokines^46^. Thus, macrophages have anti- and pro-fibrotic functions dependent on the disease phase and polarization state. For instance, deficiency for the alternative (M2) activation signature molecule Retnla leads to an ameliorated phenotype in fibrosis. A lack of Retnla results in a decreased activation of fibroblast and reduced pro-inflammatory cytokine, while overexpression augmented the fibrotic response^47^.In our study, we found increased IL-10 production in PTEN^MyKO^ mice, which could be attributed in part to alternatively activated macrophages. Further, in the lungs of PTEN^MyKO^ mice we observed an M2-like activation state of macrophages. This is indicated by the enhanced expression of Stabilin-1 and Retnla. The same trend was detected when we analysed the polarization state of alveolar macrophages derived from treatment-naive mice *in vitro*. Indeed, PTEN^MyKO^ alveolar macrophages showed enhanced expression of Retnla and Arginase I *in vitro*. However, the effect of PTEN deficiency in alveolar and interstitial macrophages on Arginase I expression in the lung is more complex. Under M2 promoting conditions such as stimulation with IL-4 and IL-13 PTEN deficiency leads to increased Arginase I expression in macrophages. However, *in vivo* during BIPF, we found slightly elevated levels of Arginase I expression in the total lung, but the percentage of CD45+ F4/80+ Arg1+ macrophages was similar in PTEN^MyKO^ compared wild-type littermates. This may indicate that other cells such as fibroblasts, myofibroblasts, epithelial or endothelial cells, contribute to the increased expression in whole lung. In essence, our observation of increased M2 marker expression and decreased numbers of extravasated macrophages in PTEN^MyKO^ mice point at beneficial effects rather than aggravation of fibrosis. In contrast, we found increased levels of pro-inflammatory cytokines in the lungs of PTEN^MyKO^ mice. This suggests that, although myeloid PTEN deficiency led to reduced macrophage recruitment into the lung, the inflammatory response was increased.

**Figure 7:**
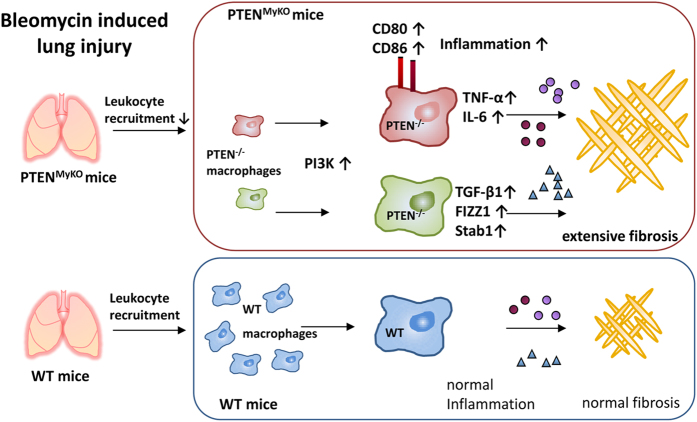
Schematic illustration of the proposed mechanism. Lung injury is induced by BLM leading to leukocyte recruitment, accompanied by inflammation and followed by fibrotic collagen deposition. Deficiency of myeloid PTEN resulted in decreased macrophage extravasation and changes in their activation and polarization state. Two phenotypes were identified in these mice during fibrosis. One subset is polarized towards an M2- like macrophages (upregulation of Stab1, FIZZ1 and TGF-β1) and the other subset is polarized towards M1 macrophages (increased expression of CD80, CD86, TNF-α and IL-6). Together, they promote increased collagen deposition and fibrosis.

Notably, we found a population of PTEN deficient macrophages, which were polarized towards a classic activation phenotype (M1). This is indicated by the significantly enhanced surface expression of CD86 and intracellular expression of iNOS and a trend towards increased CD80 expression. These findings are to some extent contradictory to the alternative activation phenotype that we observed in the PTEN^MyKO^ mice. This mixed M1/M2 phenotype may indicate that in PTEN^MyKO^ mice the inflammatory and the tissue repair phase of wound healing proceed in parallel, which is described as a hallmark of fibrosis (schematic representation in Fig. 7).The clarification of this observed effect will be subject to further investigation. Although we found a shift towards an M2 phenotype in PTEN deficient macrophages after stimulation with IL-4 and IL-13 *in vitro*, *in vivo* the situation seems to be more complex. During BIPF many additional mediators, such as TNF-α or IL-6 are released and influence macrophage polarization leading to various subpopulation in mice with myeloid PTEN deficiency. Nonetheless, the concomitant expression of TNF-α and IL-6 as well as the identification of M1-polarized macrophages led us to the conclusion that PTEN^MyKO^ mice lack important immune suppressive features and that the macrophages might be more potent effector cells resulting in exacerbated pulmonary fibrosis.

However, protection from fibrosis could be provided by T-cells. Th1 cells, for example, disclose beneficial effects on pulmonary fibrosis via the secretion of anti-fibrotic IFN-γ^48^. Controversial roles in the progression of fibrosis have been identified for regulatory T cells. It was shown that increased amounts of Tregs correlate with ameliorated idiopathic pulmonary fibrosis and Tregs can protect from TGF-β1-induced fibrosis via the release of IL-10^49^. In contrary, Tregs upregulate also TGF-β1 and thus leading to increased collagen deposition^50^. Further, it was demonstrated that the depletion of Tregs decreased the severity of silica-induced lung fibrosis^51^. In BIPF we observed a significant reduction of CD4+ Th-cells in the BALF as well as a trend for reduced CD4+ Th-cell numbers in the lung (similar numbers were observed for CD8+ T-cells) early on in the BLM induced fibrosis. Analysis of T-cells derived from fibrotic mice revealed that T-lymphocytes from PTEN^MyKO^ mice are hypo-responsive upon re-stimulation *ex vivo* compared to those from wild-types. Furthermore, we noted a reduction in the expression of Foxp3, a signature transcription factor for Treg cells, as well as a significant reduction in T-bet, which is required for the polarization of Th1-cells in the lungs of PTEN^MyKO^ mice 7d post treatment. Taken together, we speculate that not only the reduced presence of regulatory T-cells but also the lack of Th-cells, possibly Th1-cells, contribute to the pathogenesis observed in the PTEN^MyKO^ mice.

Initially, we discovered that PTEN deficiency in myeloid cells led to enhanced deposition and production of collagen in BLM treated lungs. This resulted in enhanced reorganization of alveolar-capillary structure of the lungs. The elevated collagen production was confirmed by quantitative PCR, which indicated that certain signalling processes are involved in the regulation of collagen gene expression. The enhanced ECM synthesis was paralleled by the markedly increased expression of lysyl oxidase (LOX). LOX is required to modify collagen, which is a pre-requisite for the cross-linking of collagen. Further, LOX has recently been reported to alleviate BIPF also by modulating the inflammatory response in general^52^. LOX is additionally a target in treatment of IPF and cardiac fibrosis and is a TGF-ß1 dependent gene^3^. Indeed, we found a massive upregulation of the active form of TGF-ß1 in lungs of PTEN^MyKO^ mice^53^. Downstream analysis of TGF-ß1 signalling revealed that only SMAD2 and not SMAD3 was phosphorylated, which was slightly enhanced in PTEN^MyKO^ mice. This indicates that post-translational alterations, which can be attributed to alternatively activated macrophages (such as L-proline generation by L-arginine catabolism^54^) are secondary to the changes in the collagen gene expression profile.

In summary, our study could provide convincing evidence that the myeloid cell specific deletion of the PI3K antagonist PTEN is sufficient to reduce leukocyte recruitment, promote pathogenic inflammation, drives TGF-ß1 activation and the subsequent expression of pro-fibrotic molecules. Together, these pathologic changes result in greatly enhanced pulmonary fibrosis and underscore the importance of PI3K signalling in myeloid cells in the context of chronic inflammatory diseases.

## Methods

### Mice

All experiments, including all animal investigations, were conducted according to the institutional guidelines. The Animal Care and Use Committee of the Medical University of Vienna approved all performed animal experiments (BMWF-66.009/0103-C/GT/2007, BMWF-66.009/0254-ll/3b/2013).

We crossed floxed PTEN mice with LysMCre recombinase transgenic mice and backcrossed them to a C57BL/6 background for at least eight generations. Eight to twelve week old sex-matched littermates were used for experimental approaches. PTEN^fl/fl^LysMcre+ are referred as PTEN^MyKO^ and PTEN^fl/fl^LysMcre- as wild-type littermates. Genotyping of the mice was performed by direct polymerase chain reaction of lysed murine tissue. (PTEN Primer: for CTC CTC TAC TCC ATT CTT CCC, rev ACT CCC ACC AAT GAA CAA AC; Cre Primer: for TCG CGA TTA TCT TCT ATA TCT TCA G, rev GCT CGA CCA GTT TAG TTA CCC).

### Anesthesia

Mice were anesthetized with a mixture of Ketaminol^®^ (50mg/kg) (Intervet International GmbH, Unterschleißheim, Germany) and Xylasol^®^ (5mg/kg) (Dr. E. Gräub AG, Bern, CH) for the instillation of BLM. The inhalation narcotic Forane^®^ (Isoflurane, Abott Laboratories Ltd., Queenborough, GB) was used to euthanize mice.

### Pulmonary Fibrosis Model

Pulmonary fibrosis was induced by a single intra nasal application of BLM sulfate (0.1 iu BLM/50μl/mouse). For the high dose survival experiment 0.2 iu BLM/50μl/mouse were used (Streptomyces verticillus (Calbiochem^®^, Merck KGaA, Darmstadt, Germany)).

### Histopathology and fibrosis

For histological analysis the left lung was extracted, fixed in 4% formalin and embedded in paraffin. The sections of the lung were stained with hematoxylin and eosin (Carl Roth GmbH, Karlsruhe, Germany), Sirius Red (Direct Red 80, Sigma Aldrich Inc., Vienna, Austria) or Trichrome (Carl Roth GmbH, Karlsruhe, Germany). The extent of fibrosis was graded according to the Sirius Red and Trichrome staining from 0 (no changes in the lung structure) to 5 (severe distortion of the lung architecture and large fibrous regions). The average score from all fields covering the complete lung section (average 3–4 images/mouse) was taken to determine the histological score.

Pictures were taken with an Axio Imager.Z1 microscope (Carl Zeiss Inc., Vienna, Austria).

### Lung homogenization

The right lungs were weighed, four fold of the weight PBS was added and the tissue was homogenized using 5 mm steal beads and Precellys 24 homogenizer (Bertin technologies, Aix-en-Provence, France). The homogenate was mixed with equal volumes of Greenberger Lysis Buffer with added protease inhibitor (Roche Life Sciences, Basel, Switzerland). After 20 min incubation on ice, the samples were centrifuged at 1200 rpm at 4°C for 15 min and the supernatants were used for ELISA.

### Preparation of Single Cell Suspension of Murine Lungs

Lungs were flushed with PBS via cardiac injection to remove blood from the capillaries. Subsequently, lungs were extracted and dissected into small pieces and incubated for 1 hour in RPMI, 5% fetal calf serum (FCS), 150 iu/ml collagenase I (Gibco^®^, Grand Island, NY, USA) and 50 iu/ml DNase I (Gibco^®^, Grand Island, NY, USA) at 37°C and 5% CO_2_. Cell suspensions were squeezed through a 70 μm cell strainer and centrifuged at 1250 rpm, 4°C for 10 min. Cells were resuspended in PBS/2% FCS and pressed through a 40 μm cell strainer twice. Viable cells were determined bytrypan blue and countedwith a Bürker-Türk counting chamber.

To analyse alveolar and interstitial lung macrophages, macrophages were enriched to 87% (± 5%) (data not shown) by seeding the cells of the single lung cell suspension into an untreated polystyrene plate and íncubation in RPMI, 10% FCS, 1% penicillin-streptomycin-fungizone (PSF) and 1% L-glutamine for 2h at 37°C and 5% CO_2_.Non-adherent cells and the cell culture medium were discarded, the adherent cells were washed with PBS and harvested with 2× Laemmli buffer for Western blot analysis.

### Broncho-alveolar lavage

In order to measure cytokines in the bronchoalevolar space or to isolate alveolar macrophages, the lungs were flushed via the trachea with 1 ml or 10 ml PBS, respectively. The recollected fluid was centrifuged at 1500 rpm for 10 min at room temperature. The pellet was either used for FACS analysis, resuspended in GIBCO™ RPMI Medium for culturing or mixed with 2× Laemmli buffer for Western blot analysis.

### ELISA

Concentrations of cytokines and chemokines were determined by ELISA using F8 Maxisorb Loose Nunc-Immuno Module (Thermo Scientific-Nunc A/S, Roskilde, Denmark). For measuring IL-4, IL-13, IL-17A, TGF-β1, IL-22, TNF-α the ELISA Ready-SET-Go! ^®^ Kit (eBioscience Inc., San Diego, USA) were applied and for IL-6, IL12/23 p40, IL-10 and INF-γ the Douset (R&D Systems, Minneapolis, USA) were used. The procedure was performed according to the manufacturer’s guidelines and the OD was measured at 450 nm by EL808 Ultra Microplate Reader (Bio-Tek Instruments Inc. Winooski, VT, USA).

### Hydroxyproline assay

The frozen lungs were weighed and homogenized in pre-chilled dH_2_0 by the tissue homogenizer (INULA, OMNI TH International, Kennesaw, GA, USA). The homogenate was mixed with trichloracetic acid and incubated for 20 min on ice. After centrifugation, the samples were washed in ice-cold ethanol and then the pellet dried. Next, 6 M HCl was added and incubated for 18 hours at 95°C. After cooling down the samples to room temperature, the samples were centrifuged and the supernatant was collected. 40 μl of the supernatant and the standard were mixed with 460 μl Chloramine T solution and incubated for 30 min. As final step, 500 μl Ehrlich’s reagent was added incubated at 65°C for 20 min and the OD was measured at 550 nm.

### Flow Cytometry

10^6 cells of the single lung cell suspension or the cells obtained by broncho-alveolar lavage were stained with fluorescence labelled surface antibodies for 30 min. For intracellular staining cells were treated subsequently with Fixperm (eBioscience Inc., San Diego, USA) for 1h, followed by Permbuffer (eBioscience Inc., San Diego, USA) for 10 min and then the cells were incubated with the antibodies diluted in Permbuffer for 1h. In between washing steps were performed. The following antibodies were used α-CD11b-APC, α-CD4-FITC (eBioscience Inc., San Diego, USA), α-CD45-AF647, α-CD45-PerCP, α-F4/80-FITC (BioLegendInc, London, UK), α-iNOS-PE (BD Biosciences, Franklin Lakes, NJ USA), α-ArgI-APC (R&D Systems, Minneapolis, USA), α-CD4-APC, α-CD8-PE (Immunotools GMBH, Friesoythe, Germany), α-CD80-PE, α-CD86-PE (Tonbo Bioscience, San Diego, CA, USA).

### RNA Isolation, purification, and quantitative real-time PCR

For RNA isolation the lungs were homogenized in TRIzol, RNA was isolated according to the manufacture’s protocol and the concentration was measured by NanoPhotometer^®^ (Pearl, IMPLEN, Munich, Germany). The reverse transcription was performed using the High-Capacity cDNA Reverse transcription Kits (Applied Biosystems, US). The gene expression levels were quantified by means of Fast SYBR^®^ Green Master Mix (Applied Biosystems, Life Technologies Corporation, Carlsbad, California, USA) and the PCR was performed by the StepOne™ Real-Time PCR System (Applied Biosystems, Foster City, CA, USA). The applied primer sequences are listed in Table 1.qPCR data are expressed as fold controls of the ΔΔCT values.

### *In vitro* assays

Isolated alveolar macrophages were cultured in GIBCO™ RPMI media dosed with 10% fetal calf serum, 1% PSF and 1% L-glutamine (cell culture media). 10^5 cells were stimulated with 10 ng/ml IL-4 and 10 ng/ml IL-13 for 15 min, 30 min, 60 min or overnight. Alveolar macrophages were lysed in 2× Laemmli buffer for SDS-PAGE or in TRIzol for qPCR.

Single cells of the murine lungs were incubated for 2h in cell culture media at 37°C and 5% CO_2_, subsequently the non-adherent cells were centrifuged at 1500 rpm, 5 min, room temperature and counted.10^5 cells per well were re-stimulated with plate bound α-CD3e (eBioscienceInc., San Diego, USA) for 4 days. Afterwards the cells were centrifuged again and the supernatant was used for ELISA.

### Western blot

Protein expression and phosphorylation was determined via Western blot analysis. *In vitro* cultured cells were directly harvested in 2× Laemmli buffer. The protein samples were separated according to their molecular weight via a 10% acrylamide gel and SDS-PAGE. Subsequently, the proteins were blotted to a PVDF membrane at 4°C and 150 mA. After blocking with 5% skim milk powder (Sigma-Aldrich Inc., Vienna, Austria) in PBS-Tween 20, the membranes were probed for proteins of interest. The antibodies against phospho-AKT (Ser473, D9E), phospho- GSK-3β (Ser9, 5B3), phospho-Smad2 (Ser465/467)/Smad3 (Ser423/467, D27F4), Smad2/3, AKT were purchased from Cell Signaling Technology, Inc., antibody against Arginase I (C-2) from Santa Cruz Biotechnology, INC. and anti-actin antibody was obtained from Sigma-Aldrich Inc, Vienna, Austria. The blots were developed with SuperSignal^®^ West Femto Maximum Sensitivity Substrate (Thermo Fisher Scientific, Waltham, USA) and pictures were taken with FluorChem^®^ HD2 Chemiluminesence Imager (Alpha Innotech Corp., San Leandro, CA, USA).

### Statistical analysis

The gained values were stated as boxplots indicating the median, the 1^st^- and 3^rd^ -Quartiles and min and max, or as scatter plot with indicated mean, or as bar diagram showing the mean +/− SEM. The data were evaluated with GraphPad Prism 5 software (GraphPad, San Diego, CA, USA) by the means of 2-way-ANOVA, unpaired, two tailed Student t test or Mann Whitney test, if the samples did not follow a Gaussian distribution according to the Kolmogorov-Smirnov test. Critical for significant differences was a p-value less than 0.05 for all experiments. (p-values were expressed as follows: *p < 0.05, **p < 0.01, ***p < 0.001, ****p < 0.0001). The survival was assessed with the Kaplan-Meier analysis.

## Additional Information

**How to cite this article**: Kral, J. B. *et al.* Sustained PI3K Activation exacerbates BLM-induced Lung Fibrosis via activation of pro-inflammatory and pro-fibrotic pathways. *Sci. Rep.* **6**, 23034; doi: 10.1038/srep23034 (2016).

## Supplementary Material

Supplementary Information

## Figures and Tables

**Table 1:** Sequences of the qPCR Primers.

Gene	Forward Primer	Reverse Primer
Col1a2	AGCAGGTCCTTGGAAACCTT	AAGGAGTTTCATCTGGCCCT
Col1a1	TAGGCCATTGTGTATGCAGC	ACATGTTCAGCTTTGTGGACC
Col3a1	TAGGACTGACCAAGGTGGCT	GGAACCTGGTTTCTTCTCACC
Lox	CTATGTCTGCCGCATAGGTG	GGAGGACACGTCCTGTGACT
Tgfb1	CAACCCAGGTCCTTCCTAAA	GGAGAGCCCTGGATACCAAC
Acta2	ACTGGGACGACATGGAAAAG	GTTCAGTGGTGCCTCTGTCA
Timp3	TAGACCAGAGTGCCAAAGGG	CCAGGATGCCTTCTGCAAC
Itgav	ATTCGCCGTGGACTTCTTC	TTGCCCTCCTTCTACAATCC
Retnla	CTGGATTGGCAAGAAGTTCC	CCCTTCTCATCTGCATCTCC
ArgI	GGAAAGCCAATGAAGAGCTG	GCTTCCAACTGCCAGACTGT
Chi3l3	TTTCTCCAGTGTAGCCATCCTT	TCTGGGTACAAGATCCCTGAA
Stab1	CCCTCCTTCTGCTCTGTGTC	CAAACTTGGTGTGGATGTCG
Rorc	CCGCTGAGAGGGCTTCAC	TGCAGGAGTAGGCCACATTACA
Foxp3	GCGAAAGTGGCAGAGAGGTA	TCCAAGTCTCGTCTGAAGGC
Tbx21	TCAACCAGCACCAGACAGAG	ATCCTGTAATGGCTTGTGGG
Hprt	CGCAGTCCCAGCGTCGTG	CCATCTCCTTCATGACATCTCGAG
