# Erratum: Sustained PI3K Activation exacerbates BLM-induced Lung Fibrosis via activation of pro-inflammatory and pro-fibrotic pathways

**DOI:** 10.1038/srep26048

**Published:** 2016-05-20

**Authors:** Julia Barbara Kral, Mario Kuttke, Waltraud Cornelia Schrottmaier, Birgit Birnecker, Joanna Warszawska, Christina Wernig, Hannah Paar, Manuel Salzmann, Emine Sahin, Julia Stefanie Brunner, Christoph Österreicher, Sylvia Knapp, Alice Assinger, Gernot Schabbauer

Scientific Reports 6: Article number: 23034; 10.1038/srep23034 published online: 03142016; updated: 05202016.

The Acknowledgements section and Author Contributions statement were omitted from this Article.

The Acknowledgements section should read:

“Floxed PTEN mice and LysMCre recombinase transgenic mice were kind gifts from T.W. Mak (Cancer Institute at Princess Margaret Hospital, University Health Network, Toronto, Ontario, Canada) and R. Johnson (University of California San Diego, La Jolla, CA), respectively. We want to thank Sophie Walter, Susanne Humpeler and Angela Knöbl for their great technical assistance and Michael Grusch and Elisabeth Lang for kindly providing us pSMAD2/3 and SMAD2/3 antibodies.

Financial support was provided by the Austrian Science Found (FWF) with the projects P24802 to GS and P24973 to AA and GS.”

The Author Contributions statement should read:

“J.B.K. (designed the study, performed experiments, analysed and interpreted results and wrote the manuscript), M.K., W.C.S. (performed experiments, participated in the discussion and interpretation of the data), B.B. (carried out animal experiments and helped in interpreting results), J.W. (helped in designing the study and performed experiments), C.W., H.P., M.S., E.S. and J.S.B. (were involved in data collection and animal experiments), C.Ö. (helped in planning and performing experiments), S.K., A.A. (participated in designing the study and contributed to the discussion of the research progress, interpretation of the results), G.S. (designed the study, was involved in data interpretation and discussion of the research progress, wrote the manuscript and approved the final version). All authors critically read and approved the manuscript.”

