# Supplementary material for: Sustained PI3K Activation exacerbates BLM-induced Lung Fibrosis via activation of pro-inflammatory and pro-fibrotic pathways
Source: Sci Rep. 2016 Mar 14;6:23034. doi: 10.1038/srep23034 (PMC4789787; doi:10.1038/srep23034)

ONLINE DATA SUPPLEMENT

Sustained PI3K Activation exacerbates Bleomycin-induced Lung Fibrosis via activation of pro-inflammatory and pro-fibrotic pathways

Julia Barbara Kral[1], Mario Kuttke[1], Waltraud Cornelia Schrottmaier[1], Birgit Birnecker[1], Joanna Warszawska[2,3], Christina Wernig[1], Hannah Paar[1], Emine Sahin[1], Julia Stefanie Brunner[1], Christoph Österreicher[1], Sylvia Knapp[2,3], Alice Assinger[1] and Gernot Schabbauer[1*]

[1] Institute of Physiology, Center for Physiology & Pharmacology, Medical Univ. Vienna, Austria;

[2]CEMM, Research Center for Molecular Medicine of the Austrian Academy of Sciences, Vienna, Austria

[3] Department of Internal Medicine I, Division of Infectious Diseases &Tropical Medicine, Medical Univ. Vienna, Austria

*Address correspondence to:

gernot.schabbauer@meduniwien.ac.at

Supplementary Figure Legend

Supplementary Figure I: **Myeloid PTEN deficient mice have a worsened survival at high doses of bleomycin.**

Mice with a PTEN deficiency and littermate controls were exposed to a high dose of bleomycin and were monitored for 21 days. The lack of PTEN led to a decreased survival also at high (0.2 u BLM) dose of BLM. The log-rank test was applied for statistical analysis, n=16-20.

Supplementary Figures

Sup. Figure I

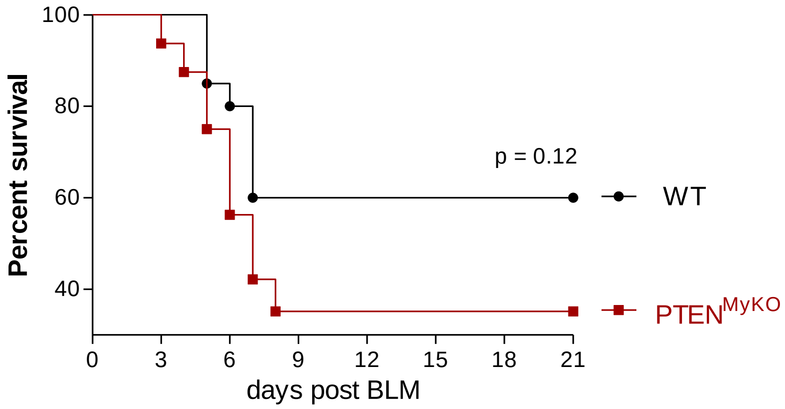

Supplement: Supplementary Information [file srep23034-s1.pdf]
